# Spatiotemporal Pattern of Neuroinflammation After Impact-Acceleration Closed Head Injury in the Rat

**DOI:** 10.1155/MI/2006/90123

**Published:** 2006-02-08

**Authors:** Servan Rooker, Sebastian Jander, Jos Van Reempts, Guido Stoll, Philippe G. Jorens, Marcel Borgers, Jan Verlooy

**Affiliations:** ^1^Department of Neurosurgery, University Hospital Antwerp, 2650 Edegem, Belgium; ^2^Department of Neurology, Heinrich-Heine University, 40225 Düsseldorf, Germany; ^3^Department of Life Sciences, Janssen Research Foundation, 2340 Beerse, Belgium; ^4^Department of Neurology, Julius-Maximilians University, 97080 Würzburg, Germany; ^5^Department of Intensive Care Medicine, University Hospital Antwerp, 2650 Edegem, Belgium

## Abstract

Inflammatory processes have been implicated in the pathogenesis of traumatic brain damage. We analyzed the spatiotemporal expression pattern of the proinflammatory key molecules: interleukin-1β, interleukin-6, tumor necrosis factor-α, and inducible nitric oxide synthase in a rat closed head injury (CHI) paradigm. 51 rats were used for RT-PCR analysis after CHI, and 18 for immunocytochemistry. We found an early upregulation of IL-1β, IL-6, and TNF-α mRNA between 1 h and 7 h after injury; the expression of iNOS mRNA only revealed a significant increase at 4 h. After 24 h, the expression decreased towards baseline levels, and remained low until 7 d after injury. Immunocytochemically, IL-1β induction was localized to ramified microglia in areas surrounding the primary impact place as well as deeper brain structures. Our study shows rapid induction of inflammatory gene expression that exceeds by far the primary impact site and might therefore contribute to tissue damage at remote sites.

## INTRODUCTION

Traumatic brain injury (TBI) is a major cause of disability, death, and economic cost to our society. In the past decades, the knowledge of the pathophysiology has remarkably increased. Primary damage to the brain is not the only important predictor for outcome but secondary events in the following hours and days may play a major role as well [1, 2]. Secondary to trauma, inflammatory processes evolve and are likely to play a major role in the evolution of brain damage. Cytokines are involved in basic neurobiological processes in the injured brain and might provide a target for pharmacological intervention [3, 4]. Proinflammatory cytokines such as tumor necrosis factor-α (TNF-α) and interleukin (IL)-1β regulate the interaction of immune and inflammatory cells, thereby orchestrating the immune response [5]. Moreover, cytokines directly act on neurons and may therefore mediate important neurotoxic as well as protective effects [3, 5–10]. Inducible nitric oxide synthase (iNOS) has been implicated as a critical downstream mediator of cytokine-induced neurotoxicity [11]. The balance between neurotoxic and protective cytokine effects may be largely determined by the time window, site, and dosage of their expression or exogenous administration both in vitro and in vivo [12].

During the past years, a rat model of CHI (closed head injury) has been developed that features several clinically relevant pathological changes, including increased ICP [13–16], disturbed autoregulation of cerebral blood flow (CBF) [17], and increased sensitivity to hypoxia [18, 19]. Recent morphologic mapping of injury in different brain areas has revealed that the piriform cortex located remote from the primary impact place at the base of the cranium is selectively vulnerable in this model of diffuse trauma (Rooker et al, unpublished data). While contrecoup forces may contribute to damage in the piriform cortex, the precise pathological mechanisms underlying such remote delayed injury in the present conditions of brain trauma are unclear. To clarify the potential role of inflammatory processes in this impact-acceleration trauma model, we have analyzed the spatiotemporal expression of IL-1β, IL-6, TNF-α, and iNOS by semiquantitative reverse transcription-polymerase chain reaction (RT-PCR) and immunhistochemistry for IL-1β.

## METHODS

### Animal experiments

Animal housing and treatment conditions complied with the European Directive 86/609 for animal welfare. A total of 69 male Sprague-Dawley rats (Charles River, Sulzfeld, Germany), weighing between 370 g and 490 g, were used for the experiments. 51 animals were used for RNA isolation and subsequent RT-PCR analysis, whereas another 18 animals were sacrificed for immunocytochemistry. Animals were allowed free access to food and water (12/12-hour day-night cycle). For the trauma experiments, the animals underwent standard anaesthesia induction with 4% isoflurane in a mixture of 30% O2 and 70% N2O over 4 minutes. Subsequently, rats were endotracheally intubated and anaesthesia was maintained with 2% isoflurane during surgical procedures.

In the sham and closed head injury (CHI) group, a 2 cm midline incision of the scalp was made and the periost removed to expose the bregma. After identification of the impact place on the bregma, rats were transferred to the trauma device as described earlier [15]. To induce CHI, a 400 g weight was dropped from a height of 50 cm. Shams underwent all of the above steps except the weight drop. After the weight drop, animals were reconnected to the gas circuit and the skull was inspected for fractures. Animals were excluded when fractures were present, otherwise the scalp was closed. When animals were spontaneously breathing again, they were included in the protocol.

### Preparation for mRNA analysis

RT-PCR analysis was performed on the following time points after trauma: 1 h (*n* = 10), 4 h (*n* = 10), 7 h (*n* = 10), 24 h (*n* = 5), 4 d (*n* = 5), and 7 d (*n* = 5). Six sham animals were used as (negative) controls. After intracerebral perfusion for 30 seconds with 0.9% NaCl under deep anesthesia, animals were sacrificed by decapitation. The brains were removed from the skull with sterile instruments and brain tissue was cut from the impact place (sample A) and the left and right piriform cortices (samples B and C, resp). Brain samples of approximately 1 mm^3^ were snap frozen in liquid nitrogen and subsequently stored at −80°C until isolation of RNA.

#### Semiquantitative RT-PCR

Total RNA was prepared from cortical tissue samples using the Trizol reagent (GIBCO BRL, Gaithersburg, Md, USA), according to the manufacturer's instructions. RNA was quantified spectrophotometrically. One microgram RNA isolated from each tissue sample was reverse transcribed using oligo (dT)20 primers and Superscript II reverse transcriptase (GIBCO BRL) essentially to the manufacturer's protocol.

cDNA equivalent to 20 ng of total RNA was subjected to subsequent PCR analysis according to a previously described protocol [20] using specific primer pairs for TNF-α, IL-1β, iNOS [21], and IL-6 (Clontech, Palo Alto, Calif, USA). Cycle numbers were 19 (GAPDH), 29 (IL-1β, IL-6), and 28 (TNF-α, iNOS). Preliminary experiments had shown that for each gene product, PCR amplification of cDNA was in the linear range under these cycling conditions (data not shown). Controls included RNA subjected to the RT-PCR procedure without addition of reverse transcriptase and PCR performed in absence of cDNA, which always yielded negative results.

### Immunocytochemistry

For the immunocytochemical analysis of IL-1β, rats were studied at 1 h, 4 h, 7 h, 16 h, 4 d, and 7 d after trauma induction (*n* = 3 in each group) according to previously established conditions [12, 22]. Rats were deeply anesthetized and perfused transcardially with 4% paraformaldehyde in 0.15 mol/L phosphate buffer (pH 7.4). Brains were removed from the skull, postfixed in the same fixative overnight, and cryoprotected by overnight infiltration with 20% sucrose in phosphate buffer at 4°C. Free floating 50 μm sections were cut on a cryostat and washed in Tris-buffered saline containing 0.05% Triton X-100 (TBS-T). Endogenous peroxidase was blocked by 30 minutes incubation in 0.3% H2O2 in TBS-T. After three washes in TBS-T, sections were incubated with affinity-purified goat antirat IL-1β polyclonal antibody (R&D Systems, Minneapolis, Minn, USA) at 0.5 μg/mL in 2% normal horse serum in TBS-T for 24 h at 4°C. After three washes in TBS-T, bound antibody was detected using biotinylated horse antigoat IgG (Vector Laboratories, Burlingame, Calif, USA) and the ABC Elite Kit (Vector) with diaminobenzidine as substrate. Control experiments in which the primary antibody was replaced by nonspecific goat IgG yielded negative results. Sections were mounted onto gelatine-coated slides, air-dried, dehydrated with ascending series of ethanol, cleared, and coverslipped with Entellan (Merck, Darmstadt, Germany).

### Statistical analysis

Statistical computations were performed using a commercially available software package for exact statistical inference (StatXact 4.0.1 for Windows). For all three investigated interleukins (IL-1β, IL-6, TNF-α) and iNOS, the normalized data sets for each survival point were compared. The groups were compared using a two-sided Wilcoxon-Mann-Whitney rank-sum test for analysis between pairs of groups separately. Two-sided probability values of less than 0.05 were regarded as statistically significant.

## RESULTS

### Time course of cytokine and iNOS mRNA expression (Figure 1)

As soon as 1 h after trauma induction, RT-PCR showed a strong increase of mRNA for IL-1β. This upregulation was not restricted to the primary impact site, but comprised the piriform cortex bilaterally as well. As compared to control rat cortex, statistically significant differences were found for all locations and on all time points up to 7 d after injury (except for sample C at 24 h (*P* = .13)). Maximal levels of mRNA were detected at 4 h after CHI. The increase remained high until 7 h and started to decline thereafter.

The expression levels of TNF-α followed the same pattern as described for IL-1β with a sharp increase as soon as 1 h after injury persisting at high levels at 4 h and 7 h after injury. Beyond 7 h mRNA levels decreased.

The expression of mRNA for IL-6 appeared to be somewhat different from the time profile as seen for IL-1β and TNF-α. In the early phase after brain injury, there was a significant rise compared with the sham animals (*P* < .01). At 4 h after injury the maximum rise was seen. From 24 h onwards, the expression levels of IL-6 mRNA already returned back to baseline levels.

Compared to the cytokines, iNOS mRNA showed a slightly more delayed induction after injury. For all three sample locations, there was no significant difference between the sham group and the group sacrificed 1 h after TBI. At 4 h, however, the maximum expression was seen.

### Time course and cellular localization of IL-1β protein expression (Figure 2)

**Figure 2:**
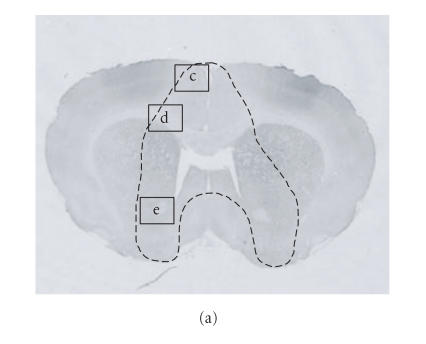

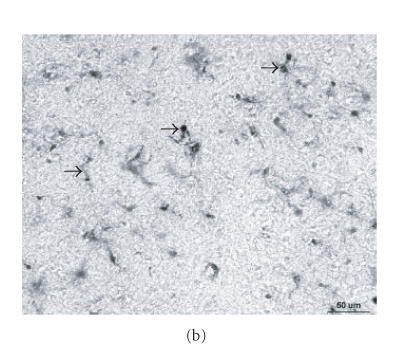

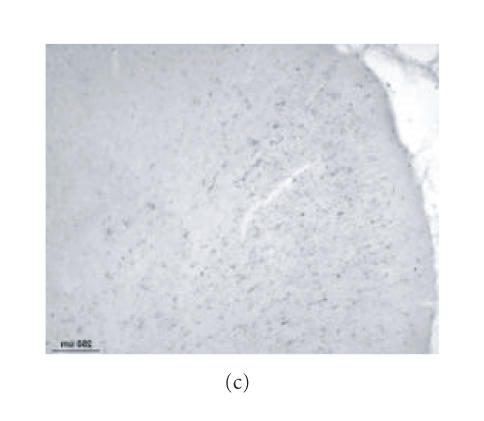

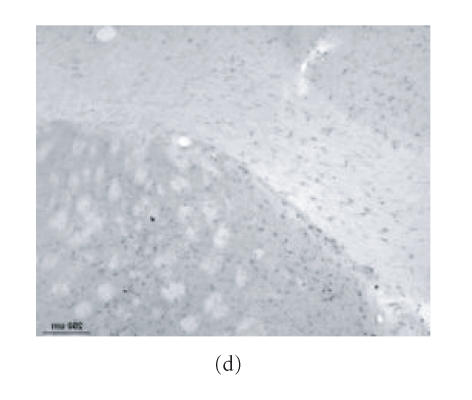

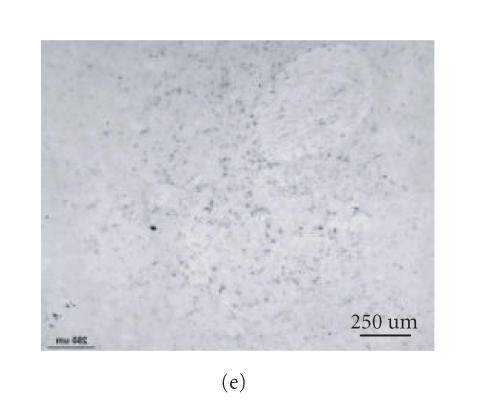
Distribution and cellular localization of IL-1β immunoreactivity 7 h after CHI. As indicated by the dashed line in (a), IL-1β expression is not restricted to the site of primary cortical injury but extends to deeper brain areas including the corpus callosum, the basal ganglia, and the piriform cortex as well. Boxed areas are displayed at higher magnification in (c), (d), and (e). (b) is a high power view showing that IL-1β-positive cells have the typical ramified morphology of resident microglia.

To analyze the expression of IL-1β protein after brain injury, we also performed immunocytochemistry with a monoclonal antibody specific for rat IL-1β. Control reactions in which the primary antibody was replaced by nonspecific goat IgG yielded negative results. IL-1β immunoreactivity was absent in the sham operated rats but the protein was clearly expressed in injured brains with peak levels reached at 7 h after trauma (Figure 2). IL-1β immunoreactivity was not restricted to the primary impact site (Figure 2(c)) but diffusely spread into deeper brain structures including subcortical white matter (Figure 2(d)), basal ganglia (Figure 2(e)), and the piriform cortex. At high magnification, IL-1β expression was localized to glial cells mostly having the typical morphology of ramified microglia (arrows in Figure 2(b)).

### Morphological changes in piriform cortex (Figure 3)

**Figure 3:**
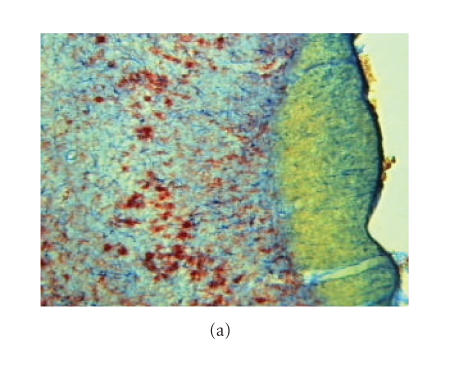

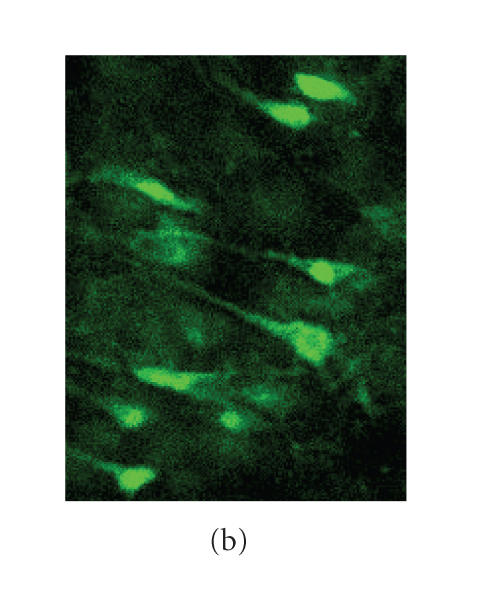

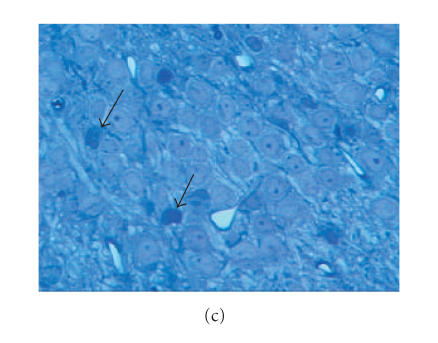
Morphological changes in the piriform cortex. Maximal microglial activation was present 7 days after moderate head injury. The microglial cells are stained with OX-42 and astrocytic cells are stained with ED-1. The depicted area is the piriform cortex, a basal structure in the rat brain (a). Neurodegeneration is shown in section (b), using fluorojade-stained vibratome sections adjacent to the ones shown in section (a). The damaged neurons can be recognized by a brightly fluorescent signal and were most numerous at 1 week after a moderate CHI. A detailed 2-micron thick epon image of the piriform cortex is shown in section (c), the arrows indicate neurons in an early stage of pycnotic degeneration.

The microglial cells stained with OX-42 and astrocytic cells stained with ED-1 appear to be maximally activated 1 week after head trauma. The depicted area is the piriform cortex, a basal structure in the rat brain (Figure 3(a)). Neurodegeneration was also most numerous at 1 week after a moderate CHI. In this study, we found that especially the piriform cortex was a structure in which microglial activation and neurodegeneration were present.

## DISCUSSION

The principal new finding of this study is that the induction of proinflammatory cytokines and iNOS in the model of CHI is not restricted to the primary impact site undergoing acute damage, but also involves the piriform cortex, a remote site undergoing delayed neurodegeneration. At both the impact site and the piriform cortex, gene induction occurred rapidly and with a similar magnitude. All cytokines studied exhibited significant increases already at 1 hour after CHI whereas the induction of iNOS was slightly more delayed reaching significant increases not before 4 hours after trauma. These data extend previous findings by other authors describing an early rise of cytokine expression but not addressing inflammatory responses at remote sites undergoing secondary degeneration [7, 9, 10, 12, 23–28].

Our mRNA findings were further substantiated by immunohistochemistry showing widespread induction of IL-1β immunoreactivity in ramified microglia throughout the brain suggesting that IL-1β expression originates from the brain parenchyma itself rather than circulating cell populations. This cellular staining pattern is in line with previous studies in focal brain ischemia identifying ramified microglia as a major source of IL-1β under pathological conditions [29]. Of note, our previous findings in a model of circumscribed cortical ischemia similarily showed that cytokine induction in brain ischemia greatly exceeds the primary injury site and additionally involves remote nonischemic cortex of the ipsilateral hemisphere. In the ischemia models, this remote cytokine induction could be blocked by the NMDA antagonist MK-801 [22] which suggests that lesion-triggered cortical spreading depression (CSD) is the main mechanism underlying remote cytokine expression in nonischemic cortex. This view has been further supported by studies in the model of KCl-induced CSD [21] if similar NMDA-dependent mechanisms underlying cytokine induction in CHI remain to be studied.

Using sensitive staining methods for neurodegeneration and microglial activation, we have found that the piriform cortex as a remote basal structure outside the primary impact place is relatively susceptible to secondary degeneration under the trauma conditions employed in the present rat model (Figure 3). Thereby, the CHI model differs from focal ischemia and CSD where neurons in remote nonischemic cortex remain completely healthy and are even protected against subsequent ischemic insults (a form of preconditioning). Of interest, iNOS mRNA in focal ischemia is always restricted to the ischemic focus and not inducible via CSD in nonischemic brain areas. Since in vitro data suggest a critical role of iNOS in the induction of neurotoxicity upon cytokine administration [11], it is tempting to speculate that the widespread induction of iNOS in piriform cortex may contribute to delayed neurodegeneration observed after CHI.

## CONCLUSIONS

In conclusion, our study shows that, in a model of diffuse head trauma, there is a rapid upregulation of proinflammatory cytokines and iNOS within hours after injury which extends from the primary injury site to the piriform cortex as a remote site undergoing delayed neurodegeneration. The piriform cortex as a well-delineated anatomical site could be an interesting area to study the role of inflammation for trauma-induced secondary neurodegeneration.

## Figures and Tables

**Figure 1:**
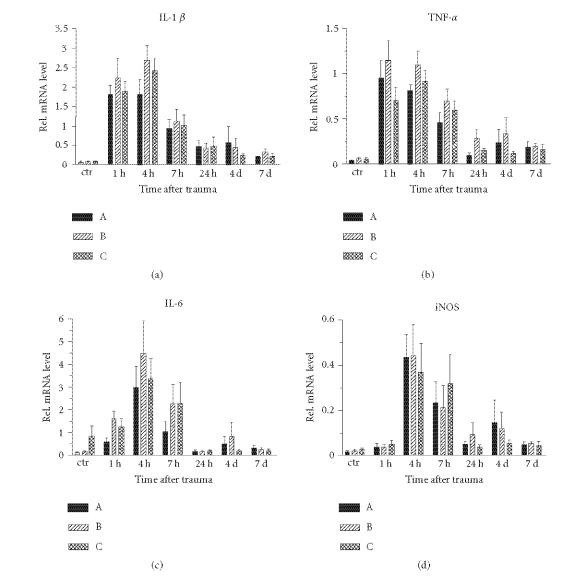
Time course of mRNA for IL-1β, TNF-α, IL-6, and iNOS in tissue sampled at the impact place (A) and the left (B) and right (C) piriform cortices. Values are expressed as mean ± SD (*n* = 10).
